# Children avoid inefficient but fair partners in a cooperative game

**DOI:** 10.1038/s41598-020-65452-9

**Published:** 2020-06-29

**Authors:** Laurent Prétôt, Gorana Gonzalez, Katherine McAuliffe

**Affiliations:** Department of Psychology, Boston College, Chestnut Hill, MA USA

**Keywords:** Social evolution, Social behaviour, Human behaviour

## Abstract

Human adults use a range of social cues to obtain information about potential partners in cooperative contexts: we prefer partners who are competent, wealthy and generous, and those who abide by moral and social rules. One factor that carries particular weight is whether a prospective partner is fair. Here we ask whether children share this preference for fair partners and, if so, whether a prospective partner’s past fair behaviour influences children’s behaviour in a cooperative dilemma. Six- to nine-year-olds chose between partners who accepted or rejected resource allocations that were either strongly advantageously unequal, strongly disadvantageously unequal, or equal. They then played a one-shot Prisoner’s Dilemma Game with their chosen partner. Children overwhelmingly preferred to play with the partner who accepted rather than rejected allocations. Regardless of their partner choice decisions, children tended to defect in the Prisoner’s Dilemma Game, yet expected that their partners would be relatively more cooperative. Finally, children were more likely to cooperate with those they believed would cooperate. Together, these findings shed new light on the links between partner choice, fairness and cooperation in child development.

## Introduction

Partner choice—whereby individuals choose or can be chosen as partners—plays a role in shaping cooperative interactions across diverse taxa^1–6^. Recent work has begun to focus on *how* partner choice decisions are made. In humans, partner choice decisions hinge on a range of behavioural cues, including generosity, morality and intentionality. For instance, adults generally choose partners who donate to others^7–10^, make deontological as opposed to utilitarian judgments^11^, and who have good intentions, even if those intentions lead to bad outcomes^12,13^.

Additionally, adults are sensitive to cues of fairness when selecting potential partners. For instance, people show a strong preference for fair partners in leadership contexts, endorsing more strongly leaders who distribute resources fairly than those who distribute unfairly^14^. Fairness even appears to outweigh other relevant information in some cases. For example, adults prefer partners who are poor but fair over those who are rich but unfair^10^. These findings suggest that partner choice and fairness may be importantly related, and indeed, some have theorized that partner choice was central to the evolution of human fairness^15^ (for a review, see^16^).

Studying partner choice in adults can teach us a great deal about the cues that partner choice decisions are sensitive to, which can, in turn, provide insight into its function. However, a focus on adults alone can teach us little about the deeper origins of partner choice. Studying the emergence of partner choice in development can provide key insight into how this behaviour is shaped over time and can shed light on the potentially deep relationship between partner choice and fairness. Here, we use a developmental approach to understand the extent to which fairness informs partner choice decisions in children and whether this has downstream consequences for children’s cooperation.

Recent work on children has started to generate insights into the developmental origins of partner choice. Children’s resource allocations often depend on the partner’s identity, including friendship, race, gender and group membership^17–20^ (for a review, see^21^). From early childhood, children are capable of evaluating agents on the basis of social actions. For example, in a study that employed a looking time paradigm in which infants evaluated geometric shapes representing agents, six- and 10-month-old infants preferred individuals who helped others over those who hindered them^22^. In a different study, 12- to 13-month-olds preferred to accept a smaller offering from a helpful agent over a larger one from a hindering agent^23^, indicating that young infants evaluate prospective partners based on their action towards others and are willing to pay a cost to interact with potentially good partners. As children age, they begin to integrate more complex information into their partner choice decisions. For instance, 13- to 17-month-old infants prefer to accept a toy from a distributor who distributes resources equally than from one who distributes resources unequally^24,25^. A preference for fair distributors is also seen in older children, but this preference is tempered by a desire for partiality in some contexts: six- to eight-year-old children prefer distributors who share resources equally between third parties in a non-competitive context but prefer those who favour them over those who are fair in a competitive context^26^.

Past work suggests that fairness behaviour may confer important information about prospective partners. However, not all responses to unfairness should be equally informative. For instance, partners who routinely prevent *themselves* from being treated unfairly (demonstrating *disadvantageous inequity aversion*) may care about fairness or they may care about improving their standing relative to others. By contrast, those who routinely prevent *others* from being treated unfairly (demonstrating *advantageous inequity aversion*) most likely care strictly about fairness. This contrast raises two important and, as yet, unanswered questions. First, based on our current understanding of the influence of fairness on partner choice, both partners described above would appear fair and would thus be equally likely to be chosen. However, the latter would likely be a better cooperative partner than the former because they adhere to fair and equal treatment even when they themselves stand to benefit from unfairness. Do children understand that some forms of fairness are more diagnostic of partner quality than others? Investigating this question can importantly inform our understanding of how fairness and partner choice are related. Second, given that fairness is expected to be important in guiding children’s partner choice decisions, do these decisions influence their cooperative behaviour and, relatedly, their beliefs about their partner’s cooperative behaviour? If partner fairness influences children’s cooperative behaviour and beliefs about their partner’s probability of cooperation, then they should cooperate more with a fair partner and less with an unfair partner. In this study, we explore these two open questions by (1) investigating children’s decisions about interacting with partners who have demonstrated disadvantageous or advantageous inequity aversion and (2) testing how these decisions influence children’s behaviour in a cooperative dilemma.

Responses to both disadvantageous and advantageous unfairness have their roots in development but show strikingly different developmental trajectories (reviewed in^27^). Young children refuse allocations that place them at a disadvantage relative to peers (*disadvantageous inequity aversion*) while older children reject both these allocations as well as allocations that place them at an advantage relative to peers (*advantageous inequity aversion*). Recent work has suggested that advantageous inequity aversion may, at least in part, serve a signalling function^28,29^. These results dovetail with other work on reputation management in children, showing that children are more prosocial, steal less, and cheat less when they are being observed by someone or think they are being observed^30–37^. Importantly, children not only become more prosocial in the presence of others, but they do so when they know they can be chosen as a potential partner, showing behaviour consistent with competitive altruism^34^.

Taken together, studies on fairness and reputation management in children suggest that, from early in development, children may advertise their fairness to potential partners in order to increase their chances of being chosen in the future. However, we currently do not know whether prospective partners actually use this information and, more specifically, whether they use it to inform their decisions about with whom to cooperate. In the current study, we ask whether children use inequity aversion as a signal of partner quality, and whether partner choice predicts their behaviour in a cooperative context.

In our study, six- to nine-year-old children learned about others’ decisions in the Inequity Game^38^. Prospective partners either accepted or rejected different types of allocations: advantageously unequal, disadvantageously unequal and equal allocations. We used a strong form of inequity aversion in both disadvantageous and advantageous contexts, one in which (1) one child received one candy while the other received four candies and (2) accepting an allocation rewarded both players while rejecting an allocation rewarded nobody. This strong marker of inequity aversion was chosen because it has been used extensively in past work on inequity aversion in children^28,38^ and we thus have a clear picture of how children of different ages, including those within the age range tested here, respond in this context. After choosing whether they wanted to partner with the acceptor or rejector, children played a one-shot Prisoner’s Dilemma Game with their chosen partner, and we solicited their beliefs about what the partner would do in the Prisoner’s Dilemma Game.

The Prisoner’s Dilemma Game, a classic game theory scenario, has long been used to explore how cooperation among adults might endure when incentives to be selfish are high^39^. In a one-shot scenario, when two players choose to *cooperate*, they maximize their combined payoff. Yet, each player has an incentive to *defect*, because defection yields a better personal payoff regardless of what the partner does. Work on this paradigm has led to a wealth of evidence concerning human cooperation in adults (for a review, see^40^) and, more recently, in children and adolescents^41–47^. In the current study, we used a manual version of the apparatus based on the recent design by Blake and colleagues^42^.

Before describing our approach in detail, it is useful to contextualize our study question and design in existing theoretical and empirical work that has examined decision-making in cooperative dilemmas. Research in evolutionary game theory has analysed the kinds of cues that support the evolution of partner choice strategies in cooperative contexts. This work has found that partner choice decisions can be usefully informed by cues like kinship^48^, past cooperativeness^49^, and, in some cases, even seemingly arbitrary cues to similarity^50^ (e.g., accent, fashion). Of particular relevance for the present study is recent work using the Prisoner’s Dilemma Game that found that wealth holdings can provide a basis for partner choice decisions in cooperative contexts: cooperation can arise when individuals choose to partner with players possessing equal wealth holdings to their own^51,52^. These findings point to a potentially important link between resource inequality, partner choice and cooperation. However, past work investigating partner choice in a game theoretic framework has largely focused on developing theoretical models or studying adult behaviour^7–13^. Our study thus offers a novel empirical contribution to this existing literature by testing whether children use fairness as a cue to inform their partner choice decisions in a cooperative context.

Our broad aim in this study was to test the hypothesis that advantageous inequity aversion is a useful signal of partner quality. This hypothesis led to three main predictions regarding children’s behaviour in our task. First, we predicted that older children (eight- to nine-year-olds) would preferentially choose partners who rejected advantageous allocations over those who rejected disadvantageous or equal allocations, while younger children (six- to seven-year-olds) would show no difference in preference across treatments. This first prediction, and our chosen age groups, were anchored in previous work indicating that advantageous inequity aversion emerges around eight years of age in the USA^38,53^. We thus expected older, but not younger, children to use information about advantageous inequity aversion when choosing partners. Second, related to our first point, we predicted that older children would be most likely to cooperate in the Prisoner’s Dilemma Game with partners who rejected advantageous allocations, while cooperation levels would not differ in younger children. Third, we predicted that children would believe that rejectors of advantageous inequity would be most likely to cooperate.

## Methods

### Participants

We tested 143 six- to nine-year-old children (75 girls) in a laboratory and in public spaces (parks and museums) in the Boston area. Children were divided into two age groups: 6- and 7-year-olds (N = 71, mean = 82.56 months ± sd = 7.31, range = 72–95 months, 38 females) and 8- and 9-year-olds (N = 72, mean = 107.18 months ± sd = 6.69, range = 96–119 months, 37 females; see Supplementary Online Material (SOM), Table S1, for sample breakdown). Additionally, we tested 22 children who were excluded due to their expressed desire to stop the study prior completion (9), lack of motivation for the food rewards (6), experimenter error (2), high winds when testing outside, which caused us to cancel our testing session (1), being outside of our age range (1), prior exposure to the study (1), missing consent form (1), and parent’s disclosure of atypical development (1). Our plan was to stop data collection once we reached our goal of 24 children (divided roughly equally between girls and boys) per condition and age group, for a total of 144 participants. We selected this sample size based on typical samples used in work on inequity aversion^28,53,54^, which often targets a sample of approximately N = 20 per cell. However, our cell sizes were inexact due to exclusions and because we invited any interested child in our age range to participate in our study once we were set up for testing on a given testing day. Parents provided written informed consent and children eight years and older provided written assent, while children of all ages provided verbal assent. All consent and experimental procedures were approved by the Institutional Review Board at Boston College (IRB protocol number 16.242). The methods were carried out in accordance with the relevant guidelines and regulations.

### Design

Children were assigned to one of three conditions: advantageous, disadvantageous or equal (see details on each condition in Inequity Game section below). Within condition, children made seven partner choice decisions in which they chose between two potential partners who either accepted or rejected a given allocation type (*partner choice* phase). After each partner choice trial, children played a one-shot Prisoner’s Dilemma Game with their chosen partner and were asked what they thought their partner would do in the game (*cooperation* phase). Note that in our study partners were not present at the time of decision (see details below). While this aspect of our design reduced the social validity of our task, a clear benefit of this method is that it helped control features of prospective partners (e.g., attractiveness, emotional reactions, nonverbal communication) that could influence children’s partner choice decisions (for a discussion, see^55^). Consequently, it allowed us to direct children’s attention to the characteristics of prospective partners that we wished to examine—i.e., whether they accepted or rejected different allocations. Fig. S1 shows the full experimental set-up.

### Inequity game

The Inequity Game is a two-player game in which an actor is paired with a recipient^38^. The actor decides whether to accept or reject different allocations of rewards between themselves and the recipient. If the actor accepts, both players get rewards. If the actor rejects, both players get nothing. Allocations in the Inequity Game are either *equal* (one reward for the actor, one reward for the recipient; 1–1), *disadvantageous* from the actor’s perspective (one reward for the actor, four rewards for the recipient; 1–4), or *advantageous* from the actor’s perspective (four rewards for the actor, one reward for the recipient; 4–1).

In order to present participants with responses of potential partners while minimizing deception in the current study, we collected a total of 42 real responses from six- to nine-year-old children who had previously played the role of actor in an Inequity Game: seven choice responses where the actor rejected the allocation and seven choice responses where the actor accepted the allocation for each of the three allocations (equal, disadvantageous, advantageous). If the participant agreed to have their decision recorded in the Inequity Game, the experimenter would ask the participant to write their initials on a small, brown paper bag prior to discreetly documenting the allocation and choice response on the back of the bag. These bags were then used as stimuli in the partner choice phase of the present study.

### Apparatus

**Figure 1:**
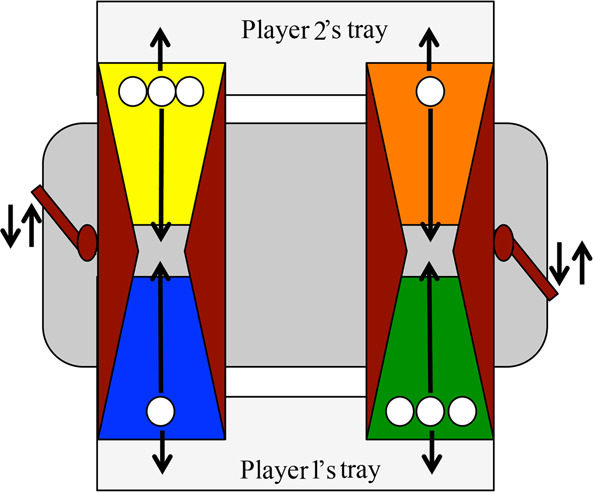
The Prisoner’s Dilemma Game apparatus consisted of two similar but independent trays (one for each player) operating in a seesaw-like fashion that delivered rewards to the players. Each player had control over one of the two trays. For instance, Player 1 would decide whether to choose the blue side (i.e., defect, as it delivers one reward to Player 1) or the yellow side (i.e., cooperate, as it delivers three rewards to Player 2) while Player 2 would decide whether to choose the orange side (i.e., defect) or the green side (i.e., cooperate).

The Prisoner’s Dilemma Game apparatus was based on the computerized task designed by Blake and colleagues^42^. We adopted the same payoffs and general structure, and then created a physical apparatus made of wood that consisted of two similar, but independent, parallel tilting trays (one for each player) with differently coloured sides (we used a combination of blue-yellow and green-orange; Fig. 1; also see SOM, Fig. S1; for other studies using this apparatus, see^56,57^). These trays worked in a seesaw-like fashion and could be tilted towards or away from the participant by pushing or pulling a handle to deliver rewards to the players or to a hidden compartment. From the perspective of the participant, pulling the handle tilted the tray towards the participant, resulting in the delivery of a single reward to the participant and the loss of the three rewards that were placed on the partner’s side of the tilting tray. This action would constitute a *defect* decision because the participant would be accepting a small payoff instead of conferring a larger payoff to their partner. By contrast, pushing the handle tilted the tray towards the partner, resulting in the delivery of three rewards to the partner and the loss of the single reward placed on the participant’s side of the tilting tray. This action would constitute a *cooperate* decision because the participant would be incurring a cost—sacrificing the single reward placed on their side—to deliver the three rewards to their partner. Thus, the task had three outcomes. If both players chose to pull the handle, which resulted in tilting the tray towards themselves, each received one reward (defect-defect). If one player pulled and the other pushed the handle, which resulted in tilting both trays towards the player who pulled only, the player who pulled received all four rewards (highest individual payoff) while the other received nothing (lowest individual payoff; defect-cooperate). Finally, if both players chose to push the handle, which resulted in tilting the tray towards the partner, each player received three rewards (highest combined payoff; cooperate-cooperate). Table 1 shows the payoff matrix used in the Prisoner’s Dilemma Game.

**Table 1:** In the Prisoner’s Dilemma Game, if the player (Player 1 or Player 2) cooperates and the partner defects, the defector receives four rewards (individual maximizing solution) and the cooperator receives none.

		*Player 2*	
		*Cooperate*	*Defect*
**Player 1**	**Cooperate**	**3**/*3*	**0**/*4*
	**Defect**	**4**/*0*	**1**/*1*

If both cooperate, each player receives three rewards (group maximizing solution), while if both defect, each player receives one reward.

### General procedure

After receiving parental consent and written assent from older children, participants were brought to a testing area that consisted of a table and the Prisoner’s Dilemma Game apparatus. Participants were given a brief overview of the study and asked for verbal assent. If the children chose to proceed, the experimenter then asked if they liked the food resource used in this study (Skittles^**®**^, a small fruit-flavoured candy), to ensure they were sufficiently motivated. Participants who reported they did not like Skittles could choose to continue with the study but were excluded from our analysis. Children were then introduced to the Prisoner’s Dilemma Game apparatus (Fig. 1). To ensure that the participants understood how the apparatus worked, the experimenter first placed one Skittle on each side of the trays, for a total of four Skittles. The experimenter then demonstrated the choices available in the game by showing the participant how choosing a particular tray (defined by its colour) would affect the delivery of resources to the child and their partner.

Participants were then asked four comprehension questions to ensure they understood the contingencies of the apparatus: (1) which colour tray would they have to choose to deliver one Skittle to themselves (*defect* option), (2) which colour tray would they have to choose to deliver one Skittle to the partner (*cooperate* option), (3) which colour trays would they and their partner have to choose to deliver two Skittles to themselves (*defect-cooperate* option), and (4) which colour trays would they and their partner have to choose to deliver two Skittles to the partner (*cooperate-defect* option). If children answered the questions incorrectly, they were given further explanations and/or demonstration and then asked again. The majority of children answered these questions correctly either spontaneously or with further explanation or demonstration (99%, 100%, 90% and 98%, respectively). In the rare cases in which a child consistently answered incorrectly, the experimenter explained the correct answer before moving on with the task.

Following the above comprehension checks, participants were presented with seven test trials, all of which showed the same kind of allocation (advantageous, disadvantageous or equal; condition was a between-subjects variable), with each trial divided in two parts: (1) the *partner choice* phase, in which participants chose one of two partners who either rejected or accepted a fair or an unfair allocation of Skittles in an Inequity Game and (2) the *cooperation* phase, in which they played a one-shot Prisoner’s Dilemma Game with the partner they had selected in the *partner choice* phase and reported whether they thought their partner would cooperate or defect. Note that the entire experimental script is available as SOM.

#### Partner choice phase

**Figure 2:**
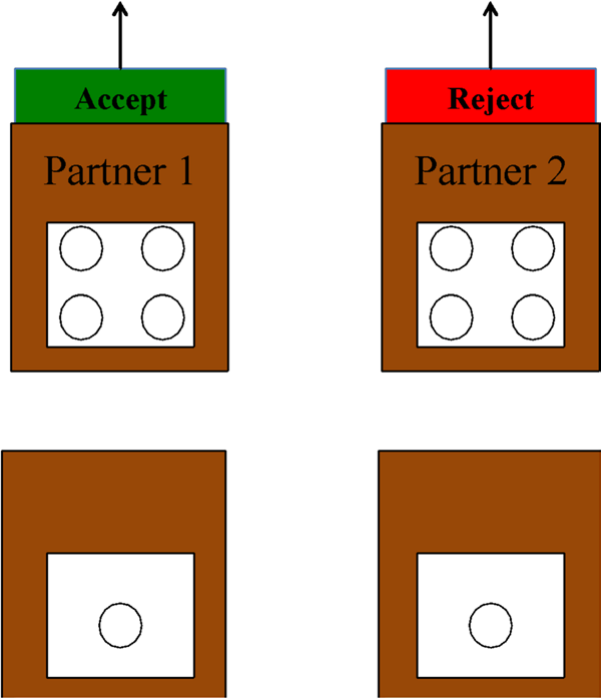
In the partner choice phase, participants learned about prospective partners’ decisions in an Inequity Game. Decisions were represented by laminated coloured cards (green for *accept* and red for *reject*), which read either “accept” or “reject”, and resource allocations were represented by laminated pictures of Skittles. Coloured cards were stored in small brown paper bags on which were written the initials of children who had previously played the inequity game, represented here by “Partner 1” and “Partner 2”.

Participants were told that they would be playing a game with other real children who also liked Skittles and who made decisions in a different game (the Inequity Game). They were then told that they could choose from these children who they would rather play with in a subsequent game (the Prisoner’s Dilemma Game), but that their chosen partner would not be present at this time. Decisions made by the other children in the Inequity Game were represented by laminated coloured cards (green for *accept* and red for *reject*), which read “accept” or “reject” at the top of the cards, and allocations were represented by laminated pictures of Skittles (Fig. 2). Participants learned that the absent children were presented with Skittles for themselves (“This kid got four Skittles”) and their partner (“This kid got one Skittle”) and that they could either *accept* the way the Skittles were divided between themselves and their partner, in which case the other children received the allotted Skittles, or *reject* the way the Skittles were divided and neither party received any Skittles to take home. The experimenter carefully explained what it meant for the other children to accept or reject allocations by placing pictures of Skittles atop the paper bag, and followed up with two comprehension questions on the first trial: (1) what happens if the partner *accepts* the resource allocation, and (2) what happens if the partner *rejects* the allocation. Following the method used for checking comprehension of the Prisoner’s Dilemma Game apparatus, if children answered these questions incorrectly, they were given further explanations and/or demonstration and then asked again. The majority of children answered these questions correctly either spontaneously or with further explanation or demonstration (97% and 98%, respectively). In the rare cases in which a child consistently answered incorrectly, the experimenter explained the correct answer before moving on with the task.

Each trial started with the experimenter revealing what two other children chose to do in the Inequity Game; in the game, one child chose to accept the allocation while the other chose to reject the allocation. To make the outcome of each decision more salient to the participants, the experimenter either moved the pictures of the Skittles into the paper bags if the child accepted the allocation (i.e., the child got to keep their Skittles and put them into their bag to take home), or removed the pictures of the Skittles out of sight if the child rejected the allocation (i.e., nobody got any Skittles to take home). Participants were then asked to choose who they would rather play with: either the kid who accepted the way the Skittles were divided or the kid who rejected the way the Skittles were divided. After each partner choice, the experimenter asked the participants to confirm their partner choice by verbalizing and pointing to their choice, at which point the partner choice phase of the trial ended. This ensured that the participants understood the decision of their partner and they were not arbitrarily pointing and choosing someone to play with. The majority of children answered this question correctly either spontaneously or with further explanation and/or demonstration in all seven trials (99–100%). In the rare cases in which a child consistently answered incorrectly, the experimenter explained the correct answer before moving on with the task. The experimenter then removed the bag of the unselected partner, and proceeded to set up the bag of the selected partner on the opposite side of the apparatus, where a real partner would have sat.

#### Cooperation phase

Following the partner choice phase, participants played one round of the Prisoner’s Dilemma Game. Each trial started with the experimenter baiting the trays with Skittles according to the Prisoner’s Dilemma Game payoff structure (Fig. 1) and asking the participants to choose a colour tray from the side of the apparatus that they controlled. The experimenter then enacted the choice of the participants, who were allowed to keep their Skittles and take them home at the end of the game. Participants were told that since the partner had not played the game yet, they would not learn about their partner’s decisions in the Prisoner’s Dilemma Game; participants would only know what their partners had done in the other game (the Inequity Game).

Before the end of each trial, participants were asked to predict what colour tray their partner would choose if they were here playing the game with them. To avoid deception, and because we were particularly interested in participants’ beliefs about their partner’s behaviour, children were told that their partner’s decision would not be enacted since their partner was not in fact present. Rather, the Skittles were removed from the partner’s side of the tray and placed into an empty bowl aside the apparatus, at which point the trial ended. To address the concern that this design may have led to a decrease in cooperation over trials, or at least after the first trial, we investigated cooperation across time but did not see strong effects.

#### Debrief questions, coding categories and responses

At the end of the procedure, the experimenter asked the child a series of post-experimental questions, including why they made the decisions they made (1) in the partner choice phase (i.e., why did they choose the partner who accepted versus rejected) and (2) in the cooperation phase (i.e., why did they choose to cooperate or defect), and (3) why they thought their partner would make the decisions that they predicted. Note that children were only asked these questions where appropriate (e.g., if a child never chose to cooperate, they were not asked why they chose to cooperate). Our primary interests were (1) whether children’s partner choices were motivated by fairness or efficiency (i.e., maximizing resources to self or other) and (2) children’s justifications for their decisions and beliefs in the Prisoner’s Dilemma Game. Consequently, we restricted our analyses to children’s responses in the partner choice phase and the cooperation phase.

For each question, children’s open-ended justifications were coded into four conceptual categories. Before coding, we had identified categories of interest based on our research questions (e.g., fairness justifications) and based on past work that has examined children’s justifications for their decisions (see^58–61^). However, before refining our coding scheme, we reviewed transcriptions of children’s responses to gain a better sense of the kinds of justifications children offered spontaneously, and then devised a coding scheme that would reliably organize their responses into meaningful categories. We were particularly interested in participants’ mentions of fairness, resources and self-maximization, and their mentions of joint- and individual resource transfers (see below for details). Each justification was included in at least one category, using a binary system (“yes” and “no”). The categories were not mutually exclusive, and a single participant’s response could be included in multiple categories. Transcriptions were made from video recordings. In cases in which we did not have a video recording or in which the child’s answer was not audible, we did not code responses.

For the partner choice phase questions (i.e., why they chose the partner who accepted or rejected), the four categories were (a) *fairness* (references to fairness, equality, wanted to be equal, even or the same; e.g., “It would be kind of fair”, “It means that everybody would get the same amount of Skittles”), (b) *resources* (references to Skittles and/or maximizing resources to self or other; e.g., “I wanted to get Skittles”, “Skittles are better than no Skittles”), (c) *other* (range of answers that were ambiguous and/or did not fit clearly into one of our other categories, excluding “I don’t know” or no answer; e.g., “I picked too much people who accepted”, “I just wanted to switch it up”), (d) *don’t know/no answer* (e.g., “I don’t know”, “I’m not sure”). The coding of open-ended responses was conducted by two coders who were blind to condition. The results of the inter-rater analyses for our two categories of interest (*fairness* and *resources*) showed that the strength of agreement was considered to be “good” to “very good” (Kappa values ranged between 0.799 and 0.961). Disagreements were resolved by discussion.

For the cooperation phase questions (i.e., why did they choose to cooperate or defect), the four categories were (a) *joint transfer* (references to transfer of resources between self and other, “we”, successful cooperation, reciprocity; e.g., “I would have gotten some of the Skittles and they would have gotten some of the Skittles”, “They got the three Skittles so then they would probably give me three Skittles”), (b) *individual transfer* (references to transfer of resources to self or other, give to other, share; e.g., “So my partner could get Skittles”, “I didn’t want them all to go to my partner’s side and not me so I let her have some”), (c) *other* (range of answers that were ambiguous and/or did not fit clearly into one of our other categories, excluding “I don’t know” or no answer; e.g., “It was my side”, “It will fall right here”), (d) *don’t know/no answer* (e.g., “I don’t know”, “I can’t remember”). The coding of open-ended responses was conducted by two coders who were blind to condition. The results of the inter-rater analyses showed that the strength of agreement for our two categories of interest (*joint transfer* and *individual transfer*) was considered to be “good” to “very good” for the cooperate question (Kappa values ranged between 0.666 and 0.877), but “poor” to “moderate” for the defect question (Kappa values ranged between 0.180 and 0.408). Therefore, we resolved disagreements for the cooperate question, and did not examine the codes from the defect question.

Note that we did not analyse responses for the other categories (i.e., “*other*” and “d*on’t know/no answer*”), because they were too vague to interpret. However, this decision likely obscured interesting variation in their responses. In particular, the “*other*” category did not make the distinction between responses that invoked social versus non-social aspects of their decisions. Future work in this area would benefit from further refining coding criteria to capture these differences.

#### Data coding and analysis

With consent, sessions were videotaped. We had three variables of interest: (1) whether children chose the partner who accepted or rejected allocations in the partner choice phase, (2) whether they cooperated or defected in the cooperation phase (Prisoner’s Dilemma Game), and (3) whether they believed their partner would cooperate or defect in the Prisoner’s Dilemma Game. Children’s decisions were live coded by the experimenter and later recoded by an independent video coder. Videos were available for 139 out of 143 participants (97%). Three videos were missing due to no consent for video recording (3) and one because the video was lost (1). To assess reliability, we compared the codes of our measures of interest between the live codes and the video codes. The results of all inter-rater analyses revealed that the strength of agreement was considered to be “very good” (all Kappa values ≥ 0.940). Disagreements between live and video coding were resolved by re-watching the video.

All statistical analyses were conducted with R statistical software (version 3.5.1)^62^. Decision data were analysed using Generalized Linear Mixed Models (GLMMs) with a binary response term (did the participant choose the partner who *rejected* (coded as 1) or *accepted* (coded as 0) in the partner choice phase, did they choose to *cooperate* (coded as 1) or *defect* (coded as 0) and did they believe their partner would *cooperate* (coded as 1) or *defect* (coded as 0) in the cooperation phase)^63^. Mixed models were run using the package ‘lme4’^64^. In all models, participant identity (ID) was fit as a random effect (intercepts) to control for repeated measures, and gender (female, male) was entered as a covariate.

Our procedure was as follows. We first created a full model, which included the predictor variables and interactions of interest. Predictors across models included condition (advantageous, disadvantageous, equal), age group (6- and 7-year-olds, 8- and 9-year-olds), partner (acceptor, rejector) in cooperation and belief models, and Prisoner’s Dilemma Game decision (cooperate, defect) in Prisoner’s Dilemma Game and belief models. We compared full models to models excluding predictors of interest using likelihood ratio tests (LRTs). We additionally compared the full model to a null model, which included only our random effect term (participant ID). In addition to reporting LRTs, we also report the Akaike Information Criterion (AIC) values associated with our models. We conducted post-hoc analyses with multivariate t (MVT) adjustments using package ‘lsmeans’. Distributional analyses were conducted using bootstrapped Kolmogorov-Smirnov tests with ‘Matching’ package. Our main models were those that fit age as a categorical term because our age groups were specifically selected based on previous work showing a shift in advantageous inequity aversion between 6- and 7-year-olds and 8- and 9-year-olds. In addition to this primary analysis, we also performed analyses with age entered as a continuous variable. Unless noted otherwise, these two approaches yielded the same general pattern of results. Finally, we used a Fisher’s test to examine children’s responses to the debrief questions, with a comparison between their mentions of fairness versus resources in the partner choice questions and joint transfer versus individual transfer in the cooperation questions (see previous section for details on each category). Barplots were created using raw data and binomial confidence intervals were calculated using the Agresti-Coull method^65^.

## Results

### Do children choose to play with those who reject advantageous unfairness?

**Figure 3:**
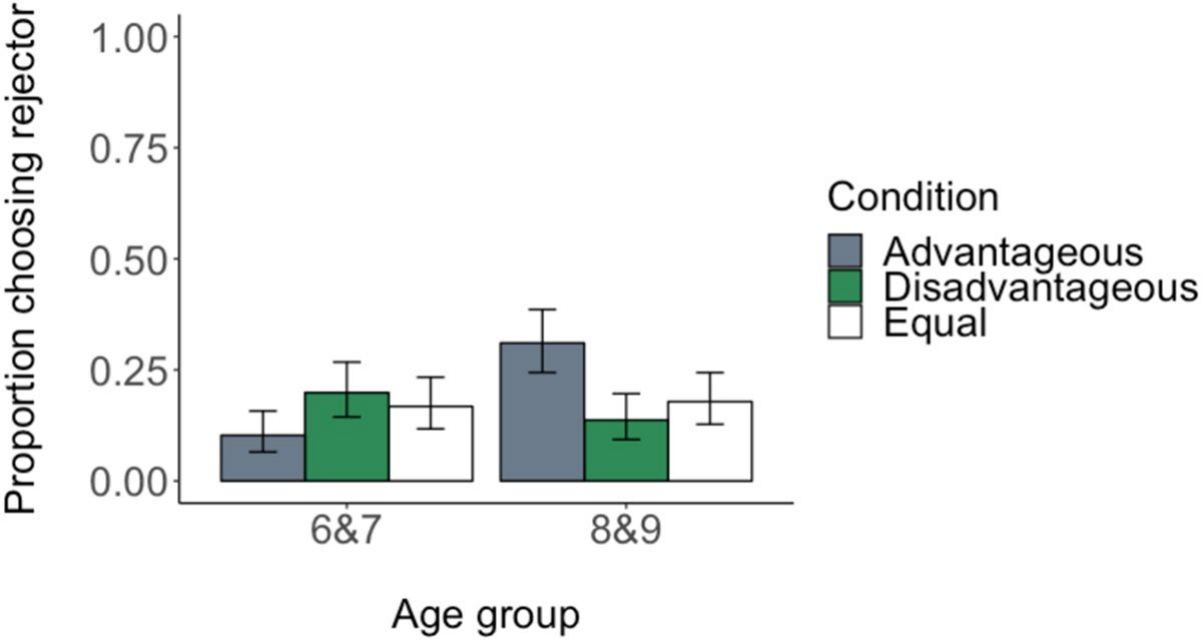
Proportion of trials in which children chose the partner who rejected an allocation across advantageous, disadvantageous and equal conditions. Proportions are shown by age group. Error bars show confidence intervals.

As Fig. 3 shows, children rarely chose rejectors, suggesting that they overwhelmingly preferred to play with partners who accepted allocations. This suggests that children wish to interact with those who have accepted resources, regardless of whether those resources were fairly or unfairly distributed, as opposed to those who have—perhaps inefficiently—rejected allocations.

#### Exploratory analyses of children’s partner choice decisions

This section consists of exploratory analyses of partner choice decisions rather than robust tests of our hypotheses. Consequently, we caution against strong interpretations of the findings from these analyses. Please note that our data and code are available to allow for further exploration.

Although children were clear in their general preference for acceptors, we nevertheless wished to examine the probability of choosing the rejector varied by condition. Here, we saw an interaction between condition and age group (LRT, χ^2^_2_ = 5.824, *p* = 0.054; Table 2). Post-hoc comparisons revealed a difference between 6- and 7-year-olds and 8- and 9-year-olds in the advantageous condition: older children were more likely to choose rejectors of advantageous inequity than were younger children (estimate = −1.702, se = 0.66, *p* = 0.01, MVT adjustment). Note, however, that our full model, which included all predictors of interest, was not a better fit to our data than the null model, which included only subject identity as random intercepts (LRT, χ^2^_6_ = 9.547, *p* = 0.145; Table 2). Indeed, a comparison of the AICs from the full and null models indicated that the null model (AIC = 820.28) better minimized information loss than the full model (AIC = 822.73). Therefore, our interaction effect of age and condition should be viewed with caution. We next turn to alternative analysis approaches to further interrogate this effect.

**Table 2:** Estimate and standard error (s.e.) of fixed effects in mixed models predicting participants’ partner choices, Prisoner’s Dilemma Game behaviour and beliefs about partner behaviour.

	Partner choice		Prisoner’s Dilemma Game behaviour			Beliefs about partner behaviour		
	Null	Full	Null	Full	Reduced	Null	Full	Reduced
Intercept	−2.39 (0.25)^***^	−3.26 (0.55)^***^	−2.26 (0.18)^***^	−2.86 (0.43)^***^	−2.72 (0.34)^***^	−0.94 (0.13)^***^	−1.06 (0.33)^**^	−1.11 (0.28)^***^
Age group: 8 & 9		1.70 (0.66)^**^		0.44 (0.57)	0.49 (0.29)		−0.62 (0.48)	−0.47 (0.26)
Condition: Disadvantageous		0.82 (0.68)		0.41 (0.56)	−0.01 (0.35)		−0.19 (0.46)	0.00 (0.31)
Condition: Equal		0.60 (0.68)		0.14 (0.57)	0.17 (0.35)		0.28 (0.45)	0.20 (0.31)
Gender: Male		0.47 (0.39)		0.18 (0.29)	0.21 (0.29)		0.07 (0.26)	0.06 (0.26)
Age group x Condition: 8 & 9 x Disadvantageous		−2.22 (0.94)^*^		−0.27 (0.78)			0.36 (0.67)	
Age group x Condition: 8 & 9 x Equal		−1.57 (0.94)		0.31 (0.78)			−0.12 (0.67)	
Partner choice: Rejector				1.21 (0.73)	0.35 (0.26)		−1.01 (0.65)	−0.23 (0.23)
Age group x Partner choice: 8 & 9 x Rejector				−0.43 (0.91)			1.14 (0.81)	
Condition x Partner choice: Disadvantageous x Rejector				−2.51 (1.17)^*^			1.17 (0.88)	
Condition x Partner choice: Equal x Rejector				−0.32 (1.01)			0.46 (0.88)	
Age group x Condition x Partner choice: 8 & 9 x Disadvantageous x Rejector				2.10 (1.44)			−1.70 (1.19)	
Age group x Condition x Partner choice: 8 & 9 x Equal x Rejector				−0.59 (1.30)			−0.61 (1.14)	
Prisoner’s Dilemma Game decision: Cooperate							2.15 (0.25)^***^	2.12 (0.25)^***^
AIC	820.28	822.73	777.24	788.21	781.39	1184.90	1122.31	1111.53
BIC	830.10	862.00	787.05	856.88	815.72	1194.71	1195.88	1150.77
Log Likelihood	−408.14	−403.37	−386.62	−380.11	−383.69	−590.45	−546.15	−547.77
Number of trials	1001	1001	997	997	997	997	997	997
Number of participants	143	143	143	143	143	143	143	143
Variance: Participant ID (Intercept)	3.57	3.12	1.36	1.38	1.25	1.33	1.37	1.35

In these models, age group was fit as a categorical predictor. Baselines for factors were: age group = 6- and 7-year-olds, condition = advantageous, gender = female, partner choice = acceptor, Prisoner’s Dilemma Game decision = defect. Table also shows goodness-of-fit statistics.

^***^p < 0.001, ^**^p < 0.01, ^*^p < 0.05.

**Figure 4:**
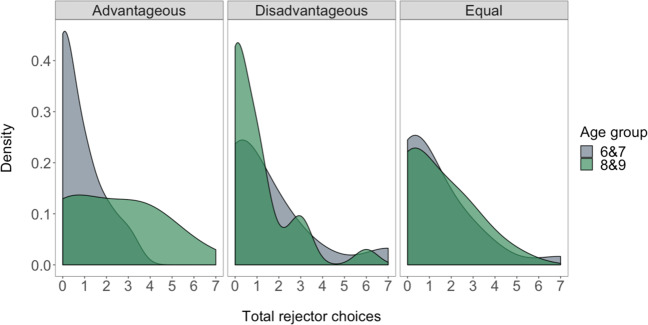
Distribution of children’s total rejector choices across advantageous, disadvantageous and equal conditions, by age group.

When we examined the distribution of children’s total rejector choices across all seven trials separately by condition (Fig. 4), we again saw a pattern indicating that older children were more likely than their younger counterparts to choose rejectors of advantageous inequality (bootstrapped two-sample Kolmogorov-Smirnov, N = 10000 repeats, D = 0.44, *p* = 0.003). The distributions between age groups did not differ in the disadvantageous or equal conditions (*p*s > 0.5).

In our secondary analysis approach, in which we fit age as a continuous predictor, we did not see a two-way interaction between age and condition (*p* = 0.152; Table S2). However, consistent with our primary analysis, this approach revealed an effect of age at the “advantageous” level of our condition term, suggesting some degree of consistency across these analyses (Table S2, Fig. S2 for predicted effects from this model).

Our partner choice data thus revealed two main findings. First, and most clearly, children were more likely to favour acceptors over rejectors regardless of condition (see intercept terms in Table 2). Second, we have converging evidence, albeit weak evidence, from different approaches that when children chose to interact with rejectors—which was rare but did happen—older children were more likely to favour rejectors in the advantageous condition than were younger children. We wish to emphasize, however, that we interpret this second result with due caution because the null model including no predictors was a better fit to the data than the model including the interaction term of interest.

### Are children more likely to cooperate with rejectors of advantageous unfairness?

**Figure 5:**
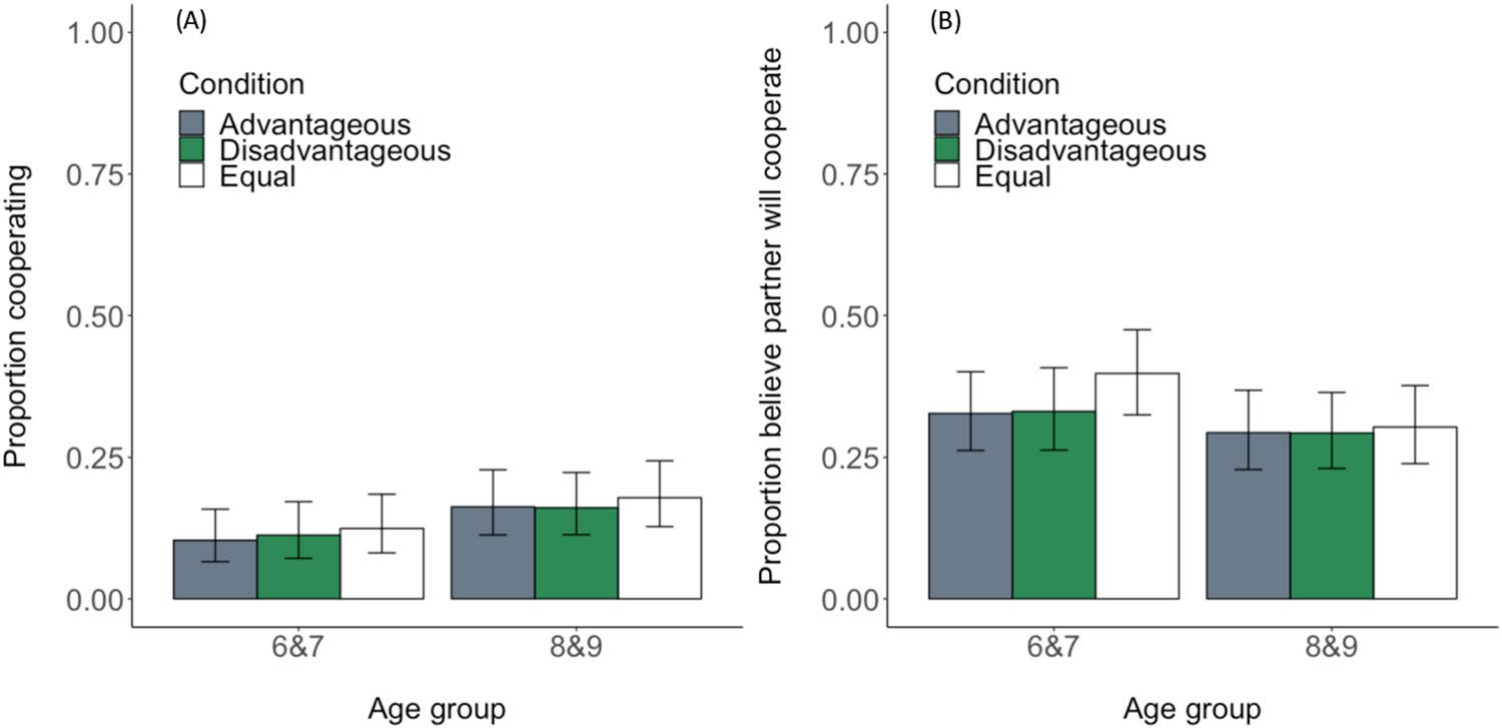
(**A**) Proportion of trials in which children cooperated in the Prisoner’s Dilemma Game across advantageous, disadvantageous, and equal conditions, and (**B**) proportion of trials in which children believed their partner would cooperate across advantageous, disadvantageous and equal conditions. Data are shown by age group. Error bars show confidence intervals.

Children were far more likely to defect in the Prisoner’s Dilemma Game than to cooperate (Fig. 5A, see intercept terms in Table 2). Contrary to our predictions, their choices in the Prisoner’s Dilemma Game were not predicted by their partner choice decisions, condition, age or interactions between predictors (*p*s > 0.1). However, our reduced model showed a marginal effect of age group on children’s cooperation, suggesting that older children were more likely to cooperate than were younger children (LRT, χ^2^_1_ = 2.966, *p* = 0.085; Table 2). As above, our full model was not a better fit to the data than a null model including only subject identity as a random effect (LRT, χ^2^_12_ = 13.029, *p* = 0.367). Supporting this, a comparison of the AICs revealed that the null model (AIC = 777.24) better minimized information loss than the full model (AIC = 788.21). Thus, our marginal effect of age group should be viewed with caution. When we ran these models with age included as a continuous predictor, we found no interaction effects (*p*s > 0.2) but again found an effect of age (LRT, χ^2^_1_ = 7.014, *p* = 0.008; Table S2).

### Do children believe that rejectors of advantageous unfairness will cooperate?

We next turn to children’s beliefs about how their chosen partner will behave in the Prisoner’s Dilemma Game. An examination of Fig. 5A relative to Fig. 5B shows that children were relatively optimistic about their partner’s behaviour: belief in partner cooperation (mean = 1.91 trials ± sd = 1.61) was higher than children’s actual cooperation in the game (mean = 0.85 trials ± sd = 1.16; paired t-test, t = −7.705, df = 142, *p* < 0.001). Belief in partner cooperation was not predicted by the three-way interaction between partner choice, age and condition, or by the two-way interaction between partner choice and condition (*p*s > 0.3). However, children’s own behaviour in the Prisoner’s Dilemma Game was a strong predictor of their belief that their partner would cooperate: if children cooperated themselves, they were more likely to believe that their partner would cooperate (Table 2). In models in which age was fit as a categorical predictor, we saw a large effect of the participant’s behaviour (LRT, χ^2^_1_ = 82.555, *p* < 0.001; Table 2). In these models, we additionally saw a marginal effect of age group: older children were slightly less likely to believe that their partner would cooperate than were younger children (LRT, χ^2^_1_ = 3.316, *p* = 0.0686; Table 2 and Fig. 5B). Our full model was better fit to the data than a null model including only subject identity as a random effect (LRT, χ^2^_13_ = 88.595, *p* < 0.001). Supporting this, a comparison of the AICs indicated that the full model (AIC = 1122.31) better minimized information loss than the null model (AIC = 1184.90). When age was fit as a continuous predictor, we again saw a large effect of the participant’s Prisoner’s Dilemma Game behaviour on their beliefs about what their partner would do (LRT, χ^2^_1_ = 82.703, *p* < 0.001, Table S2).

### Do children’s justifications invoke fairness or efficiency?

An analysis of responses to the debrief questions revealed that children referred to resources (acceptor: 68%, rejector: 39%) more often than fairness (acceptor: 11%, rejector: 19%; Fisher’s exact test, *p*s ≤ 0.029; see SOM, Table S3, for data breakdown), and to individual transfer (65%) more often than joint transfer (9%; Fisher’s exact test, *p* < 0.001; Table S3). When examining whether these responses varied as a function of conditions and age group, we found no significant effects (Fisher’s exact test, all *p*s ≥ 0.057).

## Discussion

The main result from this study was that children overwhelmingly preferred to play with partners who accepted rather than rejected allocations. This finding suggests that children avoid inefficient partners or, equally, that they value efficient partners—those who accept allocations regardless of whether or not they are fair—more than fair partners—those who reject unfair allocations. Indeed, children’s answers to debrief questions were broadly consistent with this finding: they were generally more likely to mention resources, as opposed to fairness, when explaining their decisions. With respect to children’s behaviour and beliefs in the Prisoner’s Dilemma Game, we found that children more often chose defection over cooperation, yet, they predicted that their partners would be relatively more cooperative. Finally, we found a relationship between children’s cooperative behaviour and their belief in partner cooperation, suggesting that children were more likely to cooperate with those whom they believed would cooperate.

Contrary to our original predictions, children showed an overwhelming preference for partners who accepted allocations, regardless of whether allocations were equal or unequal. Indeed, our partner choice data were further supported by children’s responses to both partner choice debrief questions and the cooperate debrief question, showing that they refer to resources and individual transfer of resources more often than fairness or joint transfer of resources. These findings are consistent with the possibility that, as partner choosers, children’s primary concern lies with ensuring that they will be paired with a partner who is willing to deliver resources. In other words, children show a preference for efficient partners rather than fair partners. This preference for efficiency could, at least in part, be explained by an aversion to choosing partners who are willing to throw candy away. Our data align with other work suggesting that children, including infants, already have a general understanding of—and expectations about—the efficiency of others’ actions. For example, three-month-old infants look longer to an agent who performs an inefficient action as compared to an efficient action^66^. In addition, 16-month-olds expect an agent to choose the more accessible of two objects^67^. Finally, by five years of age, children begin to understand that agents maximize overall utilities; that is, they take into account both the costs and rewards associated with an action, not just the rewards^68^. However, the pattern of partner choice data observed in this task is also consistent with the possibility that children *avoid* those who reject allocations. Given the set-up of the present task, we are unable to distinguish between the two possible directions of their preference—i.e., do children *prefer* acceptors or *avoid* rejectors? Future work should seek to better understand the direction of this preference.

Regardless of the underlying explanation for children’s preferences, our findings are intriguing because they are at odds with how children actually behave in the Inequity Game (reviewed in^27^), particularly when faced with advantageous inequity. If children use inequity as a public signal to others^28,29^, and do so at personal cost, why is this signal not being used more widely by receivers in a partner choice context? One possibility is that there is a mismatch between what children think potential partners expect them to do and what potential partners actually want them to do. Alternatively, children might perceive rejectors as individuals who like to destroy resources rather than create equality. However, we believe this explanation is unlikely in light of previous research showing that children would rather throw a resource away than distribute it unequally, suggesting that creating equality is their primary concern^55^. Nevertheless, future work should seek to gain a better understanding of how children perceive acceptances and rejections of inequality in this task.

One possible explanation for children’s lack of preference for fair partners is that we used too extreme a form of inequity aversion in our task. In our task, rejectors of unfairness chose to deliver no rewards as opposed to delivering a total of five rewards between themselves and a partner. We chose this form of inequity aversion because it has been widely used in past work^28,38^ and we thus have a good sense of the developmental trajectory of responses to this kind of inequity and we know that responses are, at least in part, informed by reputation concerns^28,29^. However, had we used a less extreme form of inequity aversion—for example, presenting children with a partner who chose a 2–2 split as opposed to a 4–2 split, we may have been in a better position to capture a preference for fair partners if one does indeed exist in this population. The use of our strong form of inequity also makes it difficult to distinguish between decisions made because of efficiency concerns and those made based on other distributional motives, such as the desire to maximize a minimum gain (for a discussion of disentangling different distributional preferences, see^69^). Clearly, future research is necessary to clarify the distinction between efficiency concerns and other motivations in the game.

Our data point to a *possible* effect of increased attention to fairness in partner choice decisions as children develop. Older children may have been more likely than younger children to choose rejectors of advantageous allocations. If this effect is real, it aligns with work showing that older but not younger children reject advantageous allocations when playing the game^27,38^. In this context, our observed weak interaction between age group and condition makes sense: it is not until children start to reject advantageous allocations themselves that they begin to use this information in their partner choice decisions. This result also aligns with recent work on reputation suggesting that children advertise their prosociality (for a review, see^32^) and, specifically, that children reject advantageous allocations to appear fair to others^28,29^. However, as we note in our results section, this effect was clearly weak (e.g., our null model was a better fit to the data than a model that contained the age by condition interaction) and, more importantly, we did not see the expected differences *within* age group. That is, older children were no more likely to choose a rejector of advantageous inequity than rejectors of disadvantageous inequity or equity. While we cannot draw strong conclusions from this aspect of our findings at this stage, we believe future work would benefit from further examining this potentially interesting pattern of partner choice decisions in older children.

With respect to children’s decisions in the Prisoner’s Dilemma Game, our results showed that children were much more likely to defect than cooperate in the Prisoner’s Dilemma Game, regardless of whether they chose to play with acceptors or rejectors and regardless of condition. Note that our rates of cooperation (6- and 7-year-olds chose to cooperate on 11% of trials total while 8- and 9-year-olds chose to cooperate on 17% of trials total) are situated within the range of those found in a previous study using an analogous version of the one-shot Prisoner’s Dilemma Game^42^, but generally lower than those found in studies using other measures of cooperation^70,71^, perhaps due to the more complex nature of the Prisoner’s Dilemma Game. These findings corroborate our partner choice results and further suggest that children’s primary concerns lie with gaining resources. Interestingly, children believed that partners would cooperate more than they themselves cooperated in the game, indicating that they were relatively optimistic about their partners’ behaviour. One likely explanation for this result is that children were behaving selfishly: if they believed partners would cooperate, they could defect to exploit partner cooperation. However, this result may also point to a dissociation between what children did in the game and what they thought others would do, a dissociation that aligns with other work showing a gap between judgment and behaviour^72–74^.

Children’s own behaviour in the Prisoner’s Dilemma Game was a strong predictor of their belief that their partner would cooperate; that is, if children cooperated themselves, they were more likely to believe that their partner would cooperate, suggesting that their own behaviour may influence their beliefs about others (for studies showing similar effects in adults, see^75,76^). Alternatively, this relationship may have arisen because children were more likely to cooperate if they believed their partner would cooperate. Taken together, our behaviour and belief results from the Prisoner’s Dilemma Game add to those from the partner choice decisions, suggesting that children were behaving in ways that would maximize their own resources.

It is important to note that aspects of our procedure may have influenced the inferences that can be drawn from our results. First and foremost, children made decisions in the absence of real partners. We chose this approach because it helped us control for potentially influential features of prospective partners (e.g., attractiveness, emotion) so that the only varying characteristic of partners was their behaviour in the Inequity Game. However, we cannot rule out the possibility that children would have been more likely to choose advantageous rejectors in the Inequity Game, or to cooperate in the Prisoner’s Dilemma Game, had they been paired with real partners. Second and relatedly, because we used absent partners, it is difficult to know for sure whether children viewed decisions in our task as part of meaningful social interactions. Because they overwhelmingly passed comprehension checks about the task, we are confident that they *understood* the nature of the social interactions but we cannot be as confident that they *believed* that the social interactions were real. Thus, our results should be viewed in light of the possibility that participants did not see the choices as having real social consequences in the way that we intended and future work could build on our results by testing children in a richer social context, one involving real partners or videos of prospective partners. We note, however, that evidence from previous work using the Prisoner’s Dilemma Game show that 10- and 11-year-old children cooperate with anonymous partners^42^. In addition, it is not uncommon for children to interact cooperatively with unfamiliar peers who could be considered anonymous others; for instance, when using public resources like playground toys and library books, which must be maintained for common enjoyment.

Finally, we note that our Prisoner’s Dilemma Game apparatus was based on a computerized version adapted for older children^42^, and the same apparatus has been used in other studies^56,57^. However, it has not yet been validated in the sense that we do not know how children’s behaviour in this version of the game maps onto their behaviour in other instantiations of the Prisoner’s Dilemma Game. Additionally, we want to note that the Prisoner’s Dilemma Game generally represents a relatively complex cooperative scenario. Thus, future work could fruitfully seek both to validate our version of the task and to simplify the task.

To conclude, contrary to our predictions, children did not preferentially use information about fairness when choosing partners, nor did they use this information when deciding whether to cooperate in the Prisoner’s Dilemma Game. While children routinely behave fairly when faced with inequality^27^, our work suggests that this behaviour may not strongly inform their partner choice decisions. Children in our task avoided those who rejected allocations, preferring those who accepted allocations regardless of whether they were equal or unequal. Additionally, they tended to defect rather than cooperate in the Prisoner’s Dilemma Game. These findings are consistent with the idea that children were trying to maximize their rewards in this game. Together, findings from this study represent an advance in our understanding of the link between partner choice decisions, fairness and cooperation in child development, and pave the way for future work in this area.

## Supplementary information

Supplementary Online Material for: Children avoid inefficient but fair partners in a cooperative game.

## Data Availability

The data and code are available at the following link: https://osf.io/2zc5m/?view_only=5176b81d16494050b925dfdb59274cec.
