# Author Correction: Children avoid inefficient but fair partners in a cooperative game

**DOI:** 10.1038/s41598-020-73512-3

**Published:** 2020-10-01

**Authors:** Laurent Prétôt, Gorana Gonzalez, Katherine McAuliffe

**Affiliations:** Department of Psychology, Boston College, Chestnut Hill, MA USA

Correction to: *Scientific Reports* 10.1038/s41598-020-65452-9, published online 29 June 2020

In the original version of this Article, the email address for the corresponding author Laurent Prétôt was listed as: pretot@bc.edu

Correspondence and requests for materials should instead be addressed to: laurent.pretot@gmail.com

This error has now been corrected in the PDF and HTML versions of this Article.

