# Supplementary material for: Children avoid inefficient but fair partners in a cooperative game
Source: Sci Rep. 2020 Jun 29;10:10511. doi: 10.1038/s41598-020-65452-9 (PMC7324404; doi:10.1038/s41598-020-65452-9)
Supplement: Supplementary file 1 — Supplementary Online Material for: Children avoid inefficient but fair partners in a cooperative game. [file 41598_2020_65452_MOESM1_ESM.docx]

Supplementary Online Material for: *Children avoid inefficient but fair partners in a cooperative game*

Laurent Prétôt, Gorana Gonzalez, and Katherine McAuliffe

**Table of Contents**

**Table S1.** Breakdown of subjects **2**

**Figure S1.** Photograph of the experimental setup **3**

**Figure S2.** Predicted effects of choosing rejector as a function of age x condition **4**

**Table S2.** Full model output with age as a continuous predictor **5**

**Table S3.** Responses to debrief questions by category **6**

**Experimental Script** **7**

# Table S1. Breakdown of participants by condition, age group and gender.

| Condition | Age group | **Total** | Female | Male |
| --- | --- | --- | --- | --- |
| Disadvantageous | 6&7 | **23** | 12 | 11 |
|  | 8&9 | **25** | 13 | 12 |
| Advantageous | 6&7 | **25** | 14 | 11 |
|  | 8&9 | **23** | 11 | 12 |
| Equal | 6&7 | **23** | 12 | 11 |
|  | 8&9 | **24** | 13 | 11 |


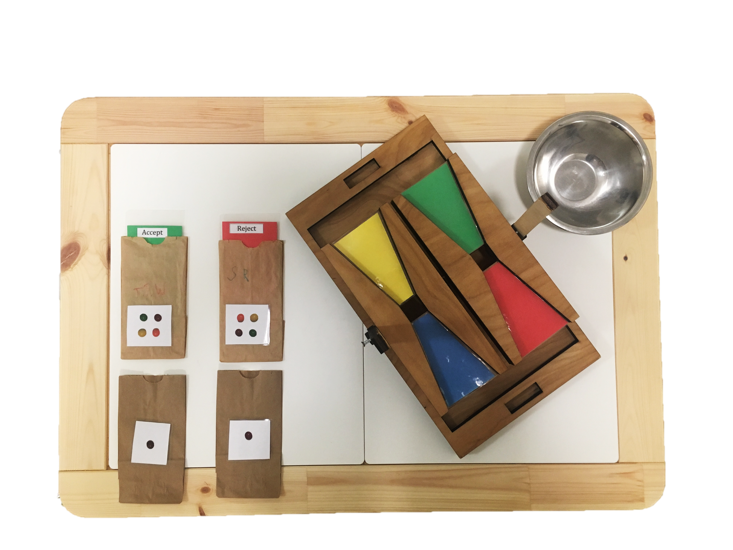


# Figure S1. Photograph of the experimental setup, including the partner choice material (left) and the Prisoner’s Dilemma Game apparatus (right).


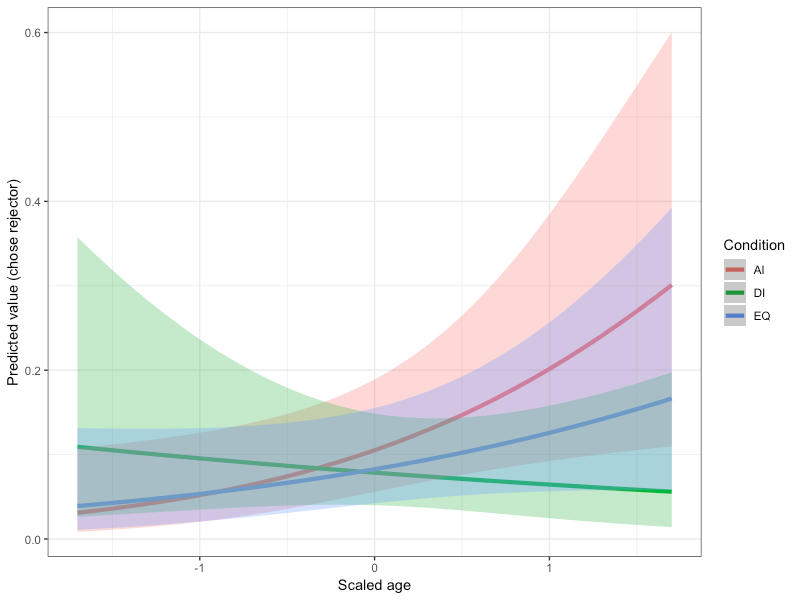


# Figure S2. Predicted effects from a model fitting the probability of choosing the rejector as a function of the two-way interaction between age and condition. AI = advantageous inequity, DI = disadvantageous inequity, EQ = equal.

# Table S2. Estimate and standard error (s.e.) of fixed effects in mixed models predicting participants’ partner choice, Prisoner’s Dilemma Game behavior, and beliefs about partner behavior. In these models, age group was fit as a continuous predictor. Baselines for factors were: gender = female, condition = advantageous, partner choice = acceptor, Prisoner’s Dilemma decision = defect. Table also shows goodness-of-fit statistics.

|  | **Partner Choice** | **Prisoner’s Dilemma Behavior** | **Beliefs about partner behavior** |
| --- | --- | --- | --- |
|  | **Full** | **Reduced** | **Reduced** |
| Intercept | -2.36 (0.41)^***^ | -2.44 (0.31)^***^ | -1.36 (0.26)^***^ |
| Gender: Male | 0.46 (0.39) | 0.20 (0.28) | 0.07 (0.26) |
| Age (scaled) | 0.76 (0.33)^*^ | 0.38 (0.14)^**^ | -0.20 (0.13) |
| Condition: Disadvantageous | -0.32 (0.47) | -0.05 (0.35) | 0.02 (0.31) |
| Condition: Equal | -0.27 (0.47) | 0.15 (0.34) | 0.20 (0.31) |
| Age x Condition (Disadvantageous) | -0.98 (0.50) |  |  |
| Age x Condition (Equal) | -0.30 (0.45) |  |  |
| Partner choice: Rejector |  | 0.32 (0.26) | -0.23 (0.24) |
| Prisoner’s Dilemma decision: Cooperate |  |  | 2.12 (0.25)^***^ |
| AIC | 821.95 | 777.34 | 1112.39 |
| BIC | 861.22 | 811.67 | 1151.63 |
| Log Likelihood | -402.97 | -381.67 | -548.19 |
| Number of trials | 1001 | 997 | 997 |
| Number of participants | 143 | 143 | 143 |
| Variance: Participant ID (Intercept) | 3.11 | 1.17 | 1.36 |
| ^***^p < 0.001, ^**^p < 0.01, ^*^p < 0.05 | | | |
|  | | | |

| Debrief question | Category | | | | | | | | | | | |
| --- | --- | --- | --- | --- | --- | --- | --- | --- | --- | --- | --- | --- |
|  | Fairness | | | Resources | | | Joint transfer | | | Individual transfer | | |
|  | Yes | No | Total | Yes | No | Total | Yes | No | Total | Yes | No | Total |
| Why chose partner who accepted? | **14** | **113** | **127** | **86** | **41** | **127** |  |  |  |  |  |  |
| *Advantageous* | 6 | 37 | 43 | 29 | 14 | 43 |  |  |  |  |  |  |
| *Disadvantageous* | 1 | 41 | 42 | 31 | 11 | 42 |  |  |  |  |  |  |
| *Equal* | 7 | 35 | 42 | 26 | 16 | 42 |  |  |  |  |  |  |
| *6&7-year-olds* | 7 | 58 | 65 | 45 | 20 | 65 |  |  |  |  |  |  |
| *8&9-year-olds* | 7 | 55 | 62 | 41 | 21 | 62 |  |  |  |  |  |  |
| Why chose partner who rejected? | **12** | **50** | **62** | **24** | **38** | **62** |  |  |  |  |  |  |
| *Advantageous* | 5 | 19 | 24 | 13 | 11 | 24 |  |  |  |  |  |  |
| *Disadvantageous* | 6 | 13 | 19 | 6 | 13 | 19 |  |  |  |  |  |  |
| *Equal* | 1 | 18 | 19 | 5 | 14 | 19 |  |  |  |  |  |  |
| *6&7-year-olds* | 4 | 27 | 31 | 15 | 16 | 31 |  |  |  |  |  |  |
| *8&9-year-olds* | 8 | 23 | 31 | 9 | 22 | 31 |  |  |  |  |  |  |
| Why chose cooperative option? |  |  |  |  |  |  | **6** | **63** | **69** | **45** | **24** | **69** |
| *Advantageous* |  |  |  |  |  |  | 1 | 26 | 27 | 19 | 8 | 27 |
| *Disadvantageous* |  |  |  |  |  |  | 1 | 19 | 20 | 15 | 5 | 20 |
| *Equal* |  |  |  |  |  |  | 4 | 18 | 22 | 11 | 11 | 22 |
| *6&7-year-olds* |  |  |  |  |  |  | 2 | 31 | 33 | 20 | 13 | 33 |
| *8&9-year-olds* |  |  |  |  |  |  | 4 | 32 | 36 | 25 | 11 | 36 |

# Table S3. Responses to debrief questions by category, condition and age group.

# Experimental Script

***Before each new participant EMPTY the disposal (tin) bowl.*** *Note: sometimes children call the orange side red.*

***Assent***

Today, we’re going to play a game where you’ll learn about other kids who played a different game and then you’ll pick who you want to play with in this game using this toy. But first, you don’t have to play this game if you don’t want to, so if you need to stop at any point, just let me know and we’ll stop.

Would you like to play this game?

Great! So in this game you’ll get some skittles to take home. So I have a very important question for you, do you like skittles? OK Great! So here’s a bag for your skittles. Which color do you like? OK, can you write your initials on the bag? *(Let children choose from 2 different colored crayons.)*

***PD***

So, like I said we’re going to play a game using this toy so I want to show you how it works.

*E places apparatus on table angled 45-degrees.*

I’ll be putting some skittles down on these tilting trays. You are in charge of what happens to the skittles on this tray [*E operates child’s tray*] and your partner is in charge of what happens to the skittles on this tray [*E operates partner’s tray*].

*Demonstrate all of this:*

And this is your box. Any skittles that fall into this box go into your bag for you to take home at the end of the game. The box on the other side is for your partner. Any skittles that fall into that box go into your partner’s bag.

*Start baiting on the child’s tray from partner’s side to child’s side.*

*Now let me show you how the trays work. I’m going to put skittles on the side that you control; one skittle on the blue side and one skittle on the yellow side. You have two choices: If you pick the **Yellow** side, the skittle on the yellow side falls into your box and the skittle on the blue side will fall into the hole and nobody will get it. Watch… [*Experimenter demonstrates this, retrieving all skittles allowing any skittles that fall in to participant’s box to be kept by the participant. “You can put your skittle(s) from your box into your bag.”*]

*Redistribute two skittles.*

OR you can pick the **Blue** side and the skittle on the blue side will fall into your partner’s box and the skittle on the yellow side will fall into the hole in the middle and nobody will get it. Watch… [*Experimenter demonstrates this, retrieving any skittles that fall into the boxes*]

Since we’re just going over the rules, I’m going to put the skittle that fell into your partners box in to this tin bowl. And the skittles that I put into this tin bowl don’t below to anyone either.

*Redistribute two skittles on the trays the partner controls.*

Just like you, your partner has two choices: they can pick the *green* side and the skittle on the *green* side will fall into your box and the skittle on the *orange* side will fall into the middle and no one gets that one. Watch… [*Experimenter demonstrates this, retrieving all skittles*]

*Redistribute the two skittles on the trays the partner controls.*

OR they can pick the *orange* side and the skittle on the *orange* side will fall into your partner’s box and the skittle on the *green* side will fall in to the middle and no one gets that one. Watch… [*Experimenter demonstrates this, retrieving all skittles*]

I know this is a little confusing so let’s do some practice to get used to the game. *Distribute 1 skittle on each color.* **What color would you choose to give one skittle to yourself?**

**And what color would your partner choose to give one skittle to themselves?** *Enact that decision. Retrieve any skittles.*

*Distribute 1 skittle on each color.*

Now I have a tricky question for you: **Which colors would you and your partner have to choose so that you get TWO skittles in your box?** *Make them say aloud and then enact the decision.*

Correct! If you were to choose color [X] and your partner were to choose color [Y] you would get two skittles.

*These will count as the comp checks. Should be coded as (1) ‘Spontaneously Correct” or if they get it wrong and need to be corrected then (2) re-explain/re-ask by re-stating the question with emphasis on the* ***you and your partner*** *and* ***TWO skittles*** *followed by pointing to their box when saying* ***in your box.*** *Code as* *“Correct with Explanation.” If they get it wrong again, move to (3) re-demonstration and indicate which trays belong to whom and whose box is whose. Then restate the question in the same manner as step 2. Code as “Correct with Demonstration.” If incorrect, (4) state correct answer and code and “Not passing” comp checks. Retrieve any skittles and place in discard bowl.*

*Distribute 1 skittle on each color.*

**And which colors would you and your partner have to choose so that your partner gets TWO skittles in their box?** *Make them say aloud and then enact the decision. Retrieve any skittles.*

Correct! If you were to choose color [X] and your partner was to choose color [Y] your partner would get two skittles.

Now it’s time for you to pick your partners!

***Partner Choice***

So to play this game *(point to apparatus)*, you have to choose which partner you’d rather play with. I’m going to tell you about a bunch of REAL kids who played a different game and who also like skittles, just like you. You’re going to learn what they decided to do in *that* game and then you’re going to choose whom you’d like to play this game with. These kids played this other game a while ago so they are not here right now. But I have the paper bags that they made and I noted down what they decided to do in the other game.

Also, since they aren’t here you won’t find out what they would do in this game. **You only know what they did in the other game.**

Let me tell you about the first set of kids who like skittles and played the other game. In the game, this kid got # skittles and their partner got # skittle. They had to decide whether to accept the way the skittles were divided, in which case they got # skittles and their partner got # skittles. Or they could reject the way the skittles were divided, in which case no gets any skittles. (*The order for mentioning, “accept” or “reject” first, is counterbalanced on the live coding sheet).*

Another kid played the same game. They got # skittles and their partner got # skittle. **Since it was the same game, what would happen if they accepted the way the skittles were divided? What would happen if they rejected the way the skittles were divided?** *(For these comprehension questions, follow the same counter balancing order for stating accept or reject first).* That’s right! If they accepted, …. And if they rejected …. *(If incorrect, re-explain, “Remember, they had to decide whether to accept the way the skittles were divided, in which case they got # skittles and their partner got # skittle. Or they could reject the way the skittles were divided, in which case no gets any skittles.” Re-ask. If incorrect a second time, re-demonstrate by flipping the cards over to show rejection of the skittle distribution. Re-ask. If incorrect a third time, state the correct response and move on to reveal the partners decisions. Code responses as “spontaneously correct”, “correct with explanation”, “correct with demonstration”, and “not passing” respectively. When re-explaining and re-demonstrating always state what happens if they REJECT or ACCEPT.)*

Ok so now I’m going to tell you what they did. This kid decided to ACCEPT/REJECT the way the skittles were divided and got # skittles and their partner got # *(demonstrate this by sliding the accept card partially out of the bag and placing the skittle cards in to the bags).* This kid REJECTED/ACCETPED the way the skittles were divided and neither of them got any skittles *(demonstrate this by sliding the accept card partially out of the bag and taking the skittles away. Children should be making their choice with only the accept and reject cards showing. The bags should be visually symmetric with nothing on them. Which partner option is presented first is counterbalanced on the live coding sheet).*

So now it’s your turn to make a decision.

*(For the 2^nd^ trial, start from this point on.)*

Who would you like to play with in this game? And what did they decide to do?

*(If children state the incorrect decision, repeat the other children’s decision (“This kid decided to accept and this kid decided to reject.”) and re-ask the line of questioning. “Who would…? And what…?” If they respond incorrectly again, re-state decisions with a full explanation and demonstration (“This kid decided to accept. That means they got X and their partner got X. This kid decided to reject. That means that no one got any skittles.”), then re-ask questions. If they respond incorrectly a third time, state the correct response and proceed with the PD part of the trial. Make a note that the child failed to respond correctly. Code responses as “spontaneously correct”, “correct with explanation”, “correct with demonstration”, and “not passing” respectively.)*

Ok so I’m going to set up their bag here… Ready to start?

**PD distribution**

*I’m going to start by putting skittles on the tray that you control. I’m going to put 3 skittles on the blue side of the tray and 1 on the yellow side of the tray.

And I’m going to put skittles on the tray that your partner controls; one the orange side and 3 on the green side.

What color do you want to choose? *(Enact the choice the child makes and state what happens. i.e. so that mean that you get one and your partner gets none.”)*

*For first trial..*

Since your partner hasn’t played this game yet, we actually don’t know what they would do. But what do you *think* they would do? *For the rest if the trials, just ask the question, “What do you think your partner would do?” DO NOT enact answers to this question. Rather just remove the skittles and place the skittles into a side bowl. You may state, for the first trial at least, “since we don’t know what they’re going to do, I’ll put these skittles into the tin bowl.”*

*If they make the mistake of choosing a color of the partner’s try, correct them and explain that they can only choose a color on their tray “Remember, you control what happens to the skittles on this tilting tray.”*

So we’re all done playing with this partner, so I’ll put their bag away. And now I’ll tell you about the next set of kids, who also like skittles.

*Move to the next partner choice.*

*This kid got # skittles and their partner got # skittle. And this kid got # skittles and their partner got # skittle. Now, let me tell you what they decided to do. *Starting with the choice on the child’s right,* this kid decided to accept/reject so… *describe and enact what happens for reject or accept…* and this kid decided to reject/accept … *describe and enact what happens for reject or accept…* Now who would you rather play with in this game? And what did they decide to do? Ok, so I’m going to set up that person’s bag. (bait the skittles.) Which color would you rather choose? *(Act out the choice)* And what color do you think your partner will choose? *(Don’t act out and place skittles in to tin bowl) Repeat this 6 times for a total of 7 trials.*

*At the end of the game….*

**When you were deciding who you’d rather play with, I noticed that sometimes you picked the kid who decide to ACCEPTED/REJECTED the way the skittles where divided. Why did you choose them?** *(Counterbalance accept rejected in parallel with which decision you introduce to the child first. If children only choose the person who accepted/rejected, only ask them about the line of choice making).**

**I also noticed that when I put skittles on the tray you control and I put three on the blue side and one on the yellow side you picked you picked the blue/yellow side. Why did you do that?** *Ask both the cooperative and defect choice if applicable.*

**I also noticed that when I put skittles on the tray your partner controls and I put one on the orange side and three on the green side you thought your partner would pick the orange/green side. Why did you do that?** *Ask both the cooperative and defect choice if applicable.*

You are all done with the game! Thank you so much for playing!
